# Naldemedine for Opioid-Induced Constipation in Patients With Cancer: A Multicenter, Double-Blind, Randomized, Placebo-Controlled Trial

**DOI:** 10.1200/JCO.24.00381

**Published:** 2024-09-10

**Authors:** Jun Hamano, Takahiro Higashibata, Takaomi Kessoku, Shinya Kajiura, Mami Hirakawa, Shunsuke Oyamada, Keisuke Ariyoshi, Takeshi Yamada, Yoshiyuki Yamamoto, Yasuyuki Takashima, Kosuke Doki, Masato Homma, Bryan J. Mathis, Tsumugi Jono, Tomoki Ogata, Kosuke Tanaka, Yuki Kasai, Michihiro Iwaki, Akiko Fuyuki, Atsushi Nakajima, Ryuji Hayashi, Takayuki Ando, Naoki Izawa, Yuko Kobayashi, Yoshiki Horie, Tatsuya Morita

**Affiliations:** ^1^ Department of Palliative and Supportive Care, Institute of Medicine, University of Tsukuba, Tsukuba, Japan; ^2^ Department of Palliative and Supportive Care, University of Tsukuba Hospital, Tsukuba, Japan; ^3^ Department of Gastroenterology and Hepatology, Yokohama City University Graduate School of Medicine, Yokohama, Japan; ^4^ Department of Gastroenterology, International University Health and Welfare Graduate School of Medicine, Narita, Japan; ^5^ Department of Palliative Medicine and Gastroenterology, International University Health and Welfare Narita Hospital, Narita, Japan; ^6^ Department of Clinical Oncology, University of Toyama, Toyama, Japan; ^7^ Department of Palliative Medicine, St Marianna University School of Medicine, Kawasaki, Japan; ^8^ Department of Biostatistics, JORTC Data Center, Tokyo, Japan; ^9^ Department of Data Management, JORTC Data Center, Tokyo, Japan; ^10^ Tsukuba Clinical Research & Development Organization (T-CReDO), University of Tsukuba, Tsukuba, Japan; ^11^ Department of Gastroenterology, Institute of Medicine, University of Tsukuba, Tsukuba, Japan; ^12^ Tsukuba Clinical Research & Development Organization, University of Tsukuba, Tsukuba, Japan; ^13^ Department of Pharmaceutical Sciences, Institute of Medicine, University of Tsukuba, Tsukuba, Japan; ^14^ Department of Cardiovascular Surgery, Institute of Medicine, University of Tsukuba, Tsukuba, Japan; ^15^ Department of Gastroenterology, Yokohama Sakae Kyosai Hospital, Yokohama, Japan; ^16^ Department of Palliative Care, Shinyurigaoka General Hospital, Kawasaki, Japan; ^17^ Third Department of Internal Medicine, University of Toyama, Toyama, Japan; ^18^ Department of Clinical Oncology, St.Marianna University School of Medicine, Kawasaki, Japan; ^19^ Department of Pharmacy, St Marianna University Hospital, Kawasaki, Japan; ^20^ Department of Palliative and Supportive Care, Seirei Mikatahara General Hospital, Hamamatsu, Japan; ^21^ Research Association for Community Health, Hamamatsu, Japan

## Abstract

**PURPOSE:**

Opioid-induced constipation is the most frequent and non–self-limiting adverse effect of opioid analgesia, reducing adherence and interfering with pain relief. This clinical trial aimed to clarify the preventive effect of naldemedine versus placebo for constipation in patients with cancer starting regularly dosed strong opioids therapy.

**METHODS:**

This multicenter, double-blinded, randomized, placebo-controlled, confirmatory trial was conducted between July 2021 and May 2023 at four academic hospitals in Japan (Japan Registry of Clinical Trials identifier: jRCTs031200397). Patients with cancer starting a first-time regularly dosed strong opioid for cancer pain and age 20+ years were included. Eligible patients were randomly assigned to the naldemedine (Symproic 0.2 mg) or placebo group in a 1:1 ratio for 14 days with protocol treatment. The primary end point was the proportion of patients with a Bowel Function Index (BFI) of <28.8 on day 14. The secondary end points included frequency of spontaneous bowel movements (SBM), quality of life (QOL), and frequency of opioid-induced nausea and vomiting (OINV).

**RESULTS:**

Of the 103 patients assessed for eligibility, 99 received either naldemedine (n = 49) or placebo (n = 50). A BFI of <28.8 on day 14 was significantly more likely to occur in the naldemedine group (64.6%; 95% CI, 51.1 to 78.1) versus placebo (17.0%; 95% CI, 6.3 to 27.8), and the difference between groups was 47.6% (95% CI, 30.3 to 64.8; *P* < .0001). The frequency of SBM, QOL, and the severity of OINV were nominally significant in the naldemedine group than in the control group.

**CONCLUSION:**

Naldemedine prevented constipation and improved constipation-related QOL, with possible preventive effect on OINV in patients with cancer starting regularly dosed opioids therapy.

## INTRODUCTION

Opioids are essential and widely prescribed for cancer pain.^1-3^ However, adverse effects, such as constipation, reduce quality of life (QOL).^4^ Opioid-induced constipation (OIC) is the most frequent and non–self-limiting adverse effect of opioid analgesia, with an estimated prevalence of up to 79%, reducing adherence and interfering with pain relief.^5-7^ Thus, bowel movement monitoring in opioid-receiving patients and coadministration of laxatives (eg, osmotic or colon-stimulating laxatives) are mandated to prevent OIC.^6-8^ However, to our best knowledge, there is no randomized placebo-controlled trial evaluating the preventive effect of laxatives for OIC.^1-3,5-9^

**CONTEXT**

**Key Objective**
To clarify the preventive effect of naldemedine versus placebo for constipation in patients with cancer starting regularly dosed therapy with strong opioids.
**Knowledge Generated**
Prophylactic naldemedine resulted in a significantly higher proportion of patients with a Bowel Function Index <28.8 and a nominally significant better quality of life on days 7 and 14 compared with placebo. The proportion of antiemetic drug use during the 72-hour period from days 1 to 3 was significantly lower with naldemedine than with placebo.
**Relevance *(C. Zimmermann)***
Preventive use of naldemedine for opioid-induced constipation may be considered, although further studies are needed to assess longer term effects and to compare with less costly alternatives.*
*Relevance section written by *JCO* Associate Editor Camilla Zimmermann, MD, PhD, FRCPC.

Recently, oral peripherally acting μ-opioid receptor antagonists (PAMORA), such as naldemedine and naloxegol, are clinically available.^10,11^ These cannot easily cross the blood-brain barrier and localize in the gut, allowing full opioid pain relief.^11^ Pathophysiologically, PAMORA-class drugs treat OIC by blocking exogenous opioids from peripheral μ-opioid receptors in the enteric nervous system submucosa and enteric plexus within the GI tract, restoring gut motility, increasing fluid secretion, and decreasing fluid absorption.^11^ Although a recent systematic review demonstrated that naldemedine manages OIC in patients with cancer on the basis of two randomized, placebo-controlled trials, evidence for prevention was absent.^12-14^ In contrast, a meta-analysis of OIC management on seven studies (n = 3,217) with prolonged-release oxycodone/naloxone for chronic noncancer pain concluded that treatment with oxycodone/naloxone reduced OIC.^15^ Furthermore, a post hoc analysis of a prospective, randomized, open-label study suggested efficacy of oxycodone/naloxone to prevent OIC in patients with opioid-naïve, chronic low back pain.^16^ There, normal Bowel Function Index (BFI) scores were significantly better maintained in the oxycodone/naloxone group versus the oxycodone and morphine groups.^16^

Thus, there are no currently reported, high-quality clinical studies confirming the preventive effect of PAMORA against OIC in patients with cancer. Additionally, animal studies demonstrated a potent and dose-dependent antiemetic effect of naldemedine against morphine-induced emetic responses. The exact mechanism remains unclear, but it is theorized that antagonizing δ-opioid receptors may play a certain role in addition to direct effects on the chemical trigger zone and improving delayed gastric emptying.^17-19^ There are also no currently reported, high-quality clinical studies on the preventive effect of PAMORA against opioid-induced nausea and vomiting (OINV) in patients with cancer. Here, we report the preventive effect of naldemedine versus placebo for constipation in patients with cancer starting an opioid therapy in a multicenter, double-blinded, randomized trial. We additionally explored the preventive effect of naldemedine against OINV.

## METHODS

### Study Design and Participants

We conducted a multicenter, double-blinded, randomized, placebo-controlled trial between July 2, 2021, and May 30, 2023, at four university hospitals in Japan.

The major inclusion criteria were patients with cancer starting a strong opioid therapy and age 20 years or older. The major exclusion criteria were patients with GI obstruction and patients who had undergone interventions affecting GI function. Detailed inclusion and exclusion criteria were previously published^20^ and are summarized in Appendix Table A1 (online only). The rationale for choosing a placebo-controlled design is summarized in Appendix 1.^9,21,22^

The Tsukuba University Clinical Research Review Board approved the protocol on January 26, 2021 (approval reference number TCRB20-001). The study was registered before enrollment in the Japan Registry of Clinical Trials (jRCT identifier: jRCTs031200397). The trial protocol was described according to Recommendations for Interventional Trials Patient-Reported Outcome Extension and its checklists.^20^ Trial results were reported according to CONSORT 2010 guidelines.^23^ Written, informed consent was obtained from all participating patients.

### Random Assignment and Masking

Eligible, consenting patients were randomly assigned to the naldemedine (Symproic 0.2 mg) or placebo group in a 1:1 ratio on the basis of a computer-generated sequence with a minimization method using institution, Eastern Cooperative Oncology Group Performance Status (≤1, ≥2), GI or non-GI cancer, and regular laxative use before enrollment (yes or no) as assignment adjustment factors.^20,24,25^ We did not include metastasis to the GI tract or peritoneum as assignment adjustment factors due to difficulties in accurate diagnoses. Allocation grouping information was available only to the data management officer on the electronic data capture (EDC) system. Research staff, patients, pharmacists, and statistical analysts were masked during the entire study medication distribution and outcome process. Masking was assessed by asking both patients and research staff to guess assignments at the end of the study to validate the double-blinding.

### Procedures

The protocol treatment period was 14 days after the start of naldemedine (or placebo). The naldemedine group received 0.2 mg Symproic once a day after breakfast for 14 days, whereas the placebo group received the placebo once a day after breakfast for 14 days. Naldemedine was purchased by the researchers from a wholesaler and supplied in capsules filled with the actual drug and lactose, whereas the placebo was supplied in capsules filled with only lactose. The first dose of oral naldemedine or placebo was given concomitantly with the first oral administration of opioids. Both groups had prescribed rescue laxatives (ie, two senna tablets) at the time of enrollment, and these could be used if a bowel movement had not occurred for over 2 days. Regular laxatives used before registration were continued without change until the end of the protocol treatment. However, for any concerns about harm (eg, frequent diarrhea), a reduction or interruption of the regular laxative dose was allowed, and such data were collected in the daily patient diary. Except for regular laxatives used before enrollment, no additional regular laxatives were used during the protocol treatment period. Rationale for use of rescue laxatives is summarized in Appendix 2.^2,3,26^

### Outcomes

Detailed end points have been published^20^ and are shown in Appendix Table A2. Among the preplanned end points, we present the primary end point, QOL measures, outcomes related to OINV, and safety.

#### Primary End Point

The primary end point was the proportion of patients with a BFI of <28.8 on day 14. The BFI is a validated, widely used three-item subjective scale specifically developed for OIC. The patients estimate their ease of defecation (0: easy/no difficulty to 100: severe difficulty), feeling of incomplete bowel evacuation (0: not at all to 100: very strong), and personal judgment of constipation (0: not at all to 100: very strong).^27^ The total score is defined as the mean of these three scores, ranging from 0 to 100, and higher scores indicate severe constipation.

This cutoff point is well established to differentiate patients with constipation from those without it.^28-30^ Although a recent formal diagnostic criterion for OIC is Rome IV, experience of its use in clinical trials is limited, whereas BFI has been used in diverse clinical trials.^31^ On the basis of the literature, we determined that BFI >28.8 was a clinically relevant constipation threshold for this study. Observation studies indicated that, to diagnose OIC on the basis of Rome IV criteria as the gold standard, the sensitivity and specificity of BFI >28.8 were 81% and 55%, respectively.^32,33^

#### Constipation-Related Outcomes

We measured the proportion of patients with a BFI of <28.8 on day 7 and the differences in BFI on days 7 and 14 versus day 1 as secondary end points. In addition to BFI, we measured the proportion of patients with three or more spontaneous bowel movements (SBM) per week and complete SBM (CSBM) per week.^11,12^ We also collected frequency data for rescue laxatives in the daily patient diary.

#### QOL Measures

We used the Japanese version of European Organization for Research and Treatment of Cancer Quality of Life Questionnaire Core 15 Palliative (EORTC QLQ-C15-PAL)^35,36^ on days 1, 7, and 14; the Patient Assessment of Constipation Quality of Life questionnaire (PAC-QOL)^37,38^ on days 1 and 14; and the Patient Assessment of Constipation Symptoms questionnaire (PAC-SYM) on days 1 and 14.^39^

#### OINV-Related Outcomes

We measured the proportion of patients who used antiemetic drugs during the 72-hour period from day 1 to day 3 and the proportion of patients who had at least one episode of vomiting during the 72-hour period from day 1 to day 3.^18^

#### Safety Outcomes

We conducted rigorous safety assessments on all adverse events occurring during the protocol treatment period using the Common Terminology Criteria for Adverse Events (CTCAE) v5.0 Japanese translation from the Japan Clinical Oncology Group.^40^ In addition, we calculated numbers and proportions of patients exhibiting CTCAE grade 3 or higher adverse events.

### Sample Size

Sample size calculation is summarized in Appendix 3.^41^

### Statistical Analyses

Efficacy and safety end point analyses were performed on the efficacy and safety analysis population as defined in the protocol paper.^20^ Primary end point comparisons of the proportion of patients with a BFI of <28.8 on day 14 between groups were conducted using a chi-square test with a two-sided significance level of 5% according to the intention-to-treat principle. Point estimates and 95% CIs were calculated for the proportion in each group and group differences.

We calculated the cumulative opioid dose during the study periods (ie, 2 weeks) from daily record in the EDC system as an oral morphine-equivalent dose according to the conversion ratio in the European Society for Medical Oncology Clinical Practice Guidelines^42^ (ie, oral morphine 60 mg = oxycodone 40 mg = hydromorphone 12 mg) and compared between the two groups with the Student *t*-test. We also compared the mean frequency of rescue laxatives per 2 weeks with the Student *t*-test.

The proportion of patients with three or more SBM per week, the proportion of patients with three or more CSBM per week on days 7 and 14, the proportion of patients who used antiemetic drugs during the 72-hour period from days 1 to 3, and the proportion of patients who had at least one episode of vomiting during the 72-hour period from days 1 to 3 were analyzed similarly to the primary end point.

Changes in BFI from day 1 to day 7 and day 14, changes in the EORTC QLQ-C15-PAL global QOL scales score from day 1 to day 7 and day 14, and the total score of PAC-QOL and PAC-SYM from days 1 to 14 were compared between groups using a two-sample t-test with point estimates, and 95% CIs of the mean and difference between groups were calculated.

For the primary end point, starting or increasing regular laxatives during protocol treatment was treated as a protocol deviation and evaluated by BFI on day 14 (not excluded from the analysis) in the primary analysis, was considered a treatment failure, and treated as equivalent to a BFI of 28.8 or higher at day 14 in the sensitivity analysis.

For safety end points, the number and incidence of all adverse events occurring during the protocol treatment period, regardless of CTCAE grade, were calculated after grading using the Japan Clinical Oncology Group shared criteria range. Additionally, the number and proportion of CTCAE grade 3 or higher events were calculated similarly. The Principal Investigator/Research Office and the Data Center reviewed and confirmed any missing, inadmissible, and abnormal data, and all statistical procedures were detailed in the statistical analysis plan before data fixation. All analyses were performed using SAS version 9.4 (SAS Institute, Cary, NC).

## RESULTS

**FIG 1.**
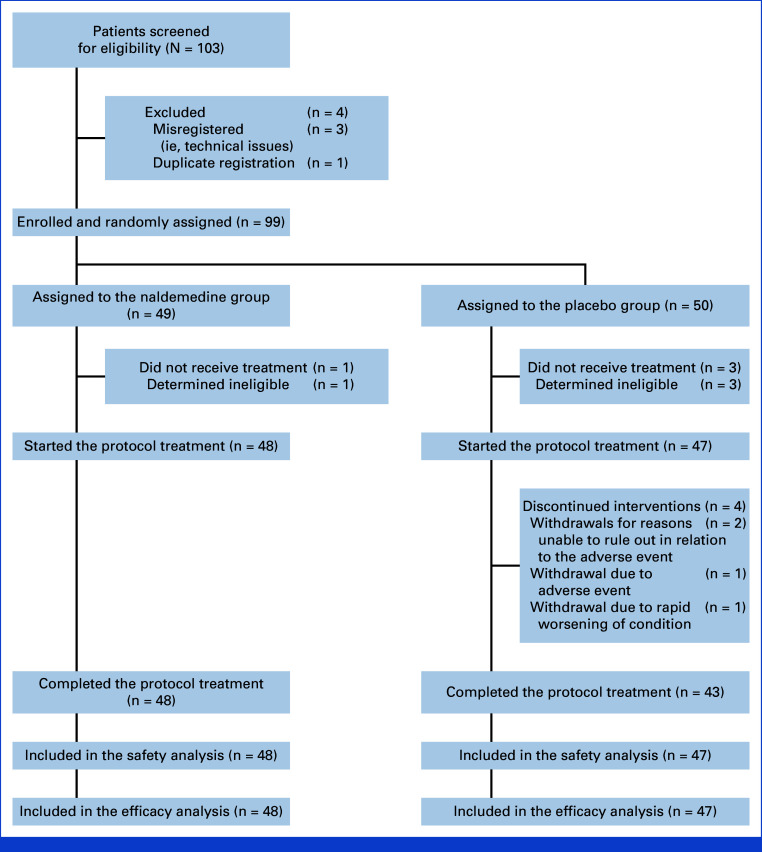
Trial profile.

Between July 2, 2021, and June 6, 2023, 103 patients were assessed for eligibility and 99 patients were randomly assigned on a 1:1 basis to receive either naldemedine (n = 49) or placebo (n = 50; Fig 1). A total of four of the 103 patients assessed for eligibility were excluded for duplicate registration (n = 1) or misregistration (ie, technical issues, n = 3). Demographic and baseline characteristics were well balanced between the two groups (Table 1). The cumulative opioid dose in the naldemedine group was significantly higher than that in the placebo group (275.7 ± 59.1 mg *v* 230.0 ± 50.7 mg; *P* = .0096).

**TABLE 1.** Patient Characteristics at Time of Enrollment

Characteristic	Naldemedine Group (n = 49)	Placebo Group (n = 50)
Median age, years	67.8 (33-84)	66.4 (32-85)
Sex, No. (%)		
Male	24 (49.0)	16 (32.0)
Female	25 (51.0)	34 (68.0)
Clinical setting at time of enrollment, No. (%)		
Hospitalization	9 (18.4)	11 (22.0)
Outpatient	40 (81.6)	39 (78.0)
Cancer type, No. (%)		
Hepatobiliary and pancreatic	18 (36.7)	17 (34.0)
GI	8 (16.3)	10 (20.0)
Gynecological	5 (10.2)	5 (10.0)
Lung	4 (8.2)	3 (6.0)
Breast	4 (8.2)	4 (8.0)
Genitourinary	4 (8.2)	4 (8.0)
Other	6 (2.0)	7 (14.0)
ECOG PS, No. (%)		
0	15 (30.6)	16 (32.0)
1	25 (51.0)	25 (50.0)
2	8 (16.3)	6 (12.0)
3	1 (2.0)	3 (6.0)
4	0	0
Metastatic site, No. (%)		
No metastases	24 (49.0)	23 (46.0)
Present	24 (49.0)	27 (54.0)
Lung	11 (22.4)	9 (18.0)
Liver	9 (18.4)	8 (16.0)
Bone	10 (20.4)	8 (16.0)
CNS	0	0
Peritoneal dissemination	3 (6.1)	10 (20.0)
Other	10 (20.4)	14 (28.0)
History of GI tract surgery, No. (%)		
No history	35 (71.4)	38 (76.0)
Yes	14 (28.6)	12 (24.0)
Strong opioids used before enrollment as rescue use only, No. (%)		
No history of use	38 (77.6)	38 (76.0)
Yes	11 (22.4)	12 (24.0)
Oxycodone	11 (22.4)	11 (22.0)
Morphine	0	0
Hydromorphone	0	0
Weak opioids used before enrollment, No. (%)		
No history of use	42 (85.7)	43 (86.0)
Yes	7 (14.3)	7 (14.0)
Tramadol	7 (14.3)	7 (14.0)
Others	1 (2.0)	0
Strong opioids starting at enrollment, No. (%)		
Oxycodone	42 (85.7)	44 (88.0)
Morphine	0	0
Hydromorphone	7 (14.3)	6 (12.0)
Laxatives used before enrollment, No. (%)		
No history of use	32 (65.3)	35 (70.0)
Yes	17 (34.7)	15 (30.0)
Magnesium oxide	16 (32.7)	8 (16.0)
Sennoside	3 (6.1)	4 (8.0)
Lubiprostone	1 (2.0)	1 (2.0)
Other	2 (4.1)	3 (6.0)
BFI		
Mean ± SD	18.3 ± 19.8	18.2 ± 20.0
BFI <28.8		
Number of patients, No. (%)	35 (71.4)	37 (74.0)
Point estimate of the percentage (95% CI)	71.4 (58.8 to 84.1)	74.0 (61.8 to 86.2)

Abbreviations: BFI, Bowel Function Index; ECOG PS, Eastern Cooperative Oncology Group performance status; SD, standard deviation.

### Primary End point

**FIG A1.**
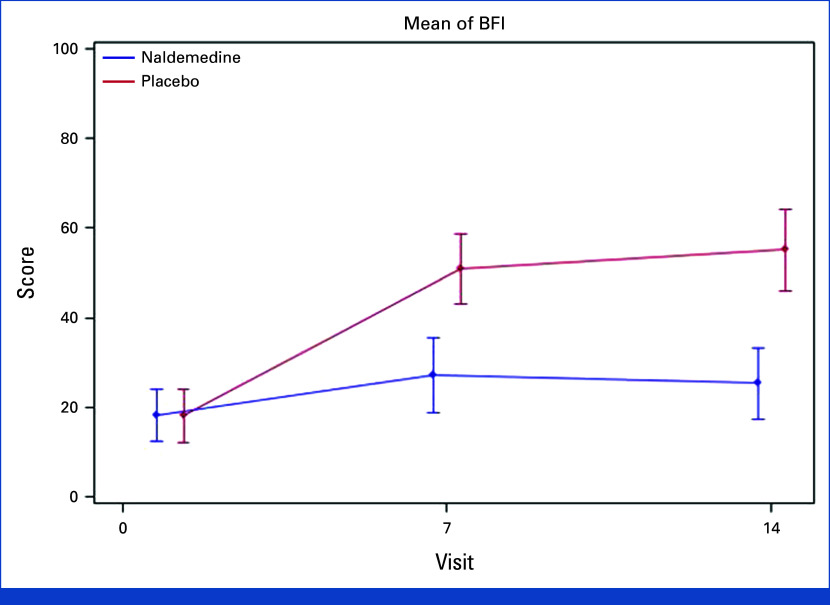
Line plot of mean BFI for naldemedine group and placebo group at days 1, 7, and 14. Bar indicated the mean of BFI with 95% CI. BFI, Bowel Function Index.

The proportion of patients with a BFI of <28.8 on day 14 was significantly greater with naldemedine (64.6% [31 of 48 patients]; 95% CI, 51.1 to 78.1) than with placebo (17.0% [eight of 47 patients]; 95% CI, 6.3 to 27.8), with a difference of 47.6% (95% CI, 30.3 to 64.8; *P* < .0001; Table 2). Changes in BFI scores during the study periods are plotted in Appendix Fig A1. The use frequency of rescue laxatives during the study period was 0.6 ± 1.5 and 2.0 ± 3.0 times/14 days in the naldemedine and placebo group, respectively (*P* = .0059; Appendix Table A3).

**TABLE 2.** The Change in Intergroup Differences of the Proportion of Patients With a BFI of <28.8, With Three or More SBM/CSBM per Week, and in EORTC QLQ-C15-PAL, PAC-SYM, and PAC-QOL Results on Days 7 and 14

End Point	Day 7				Day 14			
	Naldemedine Group (n = 48)	Placebo Group (n = 47)	Difference Between Groups (naldemedine-placebo)	*P*	Naldemedine Group (n = 48)	Placebo Group (n = 47)	Difference Between Groups (naldemedine-placebo)	*P*
BFI <28.8								
Number of patients	28	8			31	8		
Point estimate of the percentage (95% CI)	58.3 (44.4 to 72.3)	17.0 (6.3 to 27.8)	41.3 (23.7 to 58.9)	<.0001	64.6 (51.1 to 78.1)	17.0 (6.3 to 27.8)	47.6 (30.3 to 64.8)	<.0001
BFI	27.2 ± 28.8	50.9 ± 25.1			25.4 ± 27.1	55.1 ± 29.5		
Difference from day 1	9.8 (1.4-18.2)	33.1 (22.1-44.2)	–23.4 (–36.9 to –9.8)	.0011	7.1 (–0.2 to 14.3)	38.5 (26.6 to 50.4)	–31.5 (–44.9 to –18.0)	<.0001
SBM 3 or more per week								
Number of patients	41	24			42	25		
Point estimate of the percentage (95% CI)	85.4 (75.4 to 95.4)	51.1 (36.8 to 65.4)	34.4 (16.9 to 51.8)	.0003	87.5 (78.1 to 96.9)	53.2 (38.9 to 67.5)	34.3 (17.3 to 51.4)	.0002
CSBM 3 or more per week								
Number of patients	35	17			34	17		
Point estimate of the percentage (95% CI)	72.9 (60.4 to 85.5)	36.2 (22.4 to 49.9)	36.8 (18.1 to 55.4)	.0003	70.8 (58.0 to 83.7)	36.2 (22.4 to 49.9)	34.7 (15.9 to 53.5)	.0007
EORTC QLQ-C15-PAL								
Global QOL	69.1 (25.5)	69.6 (20.8)			73.2 (23.3)	71.4 (19.3)		
Difference from day 1	16.0 (4.5-27.5)	–3.2 (–12.1 to 5.7)	19.1 (4.5-33.7)	.0108	15.6 (3.4-27.8)	–2.0 (–13.6 to 9.6)	17.6 (0.9-34.3)	.0390
PAC-QOL	NA	NA			22.7 (14.4)	36.0 (19.1)		
Difference from day 1	NA	NA	NA		0.3 (–4.2 to 4.9)	11.8 (4.6-19.0)	–11.5 (–19.6 to –3.3)	.0083
PAC-SYM	NA	NA			7.7 (8.4)	16.2 (9.9)		
Difference from day 1	NA	NA	NA		0.2 (–2.5 to 2.8)	7.4 (3.0-11.7)	–7.2 (–12.0 to –2.3)	.0044

Abbreviations: BFI, Bowel Function Index; CSBM, complete SBM; EORTC QLQ-C15-PAL, European Organization for Research and Treatment of Cancer Quality of Life Questionnaire Core 15 Palliative; NA, not available; PAC-QOL, Patient Assessment of Constipation Quality of Life questionnaire; PAC-SYM, Patient Assessment of Constipation Symptoms questionnaire; SBM, spontaneous bowel movements.

In the sensitivity analysis, where treatment failure patients were considered equivalent to a BFI of 28.8 or higher at day 14, the naldemedine group had a significantly greater proportion of patients with a BFI of 28.8 or less compared with the placebo group (64.6% [31 of 48 patients] *v* 14.9% [7 of 47 patients]; *P* < .0001).

### Secondary End Points

#### BFI on Day 7

The proportion of patients with a BFI of <28.8 on day 7 was significantly greater with naldemedine than with placebo (Table 2). The differences in BFI scores at day 7 from day 1 were significantly lower with naldemedine than with placebo as well as at day 14 from day 1.

#### SBM and CSBM

The proportion of patients with three or more SBM per week and CSBM per week on day 7 was significantly greater in the naldemedine group than in the placebo group (Table 2). Results from day 14 also revealed significantly greater proportions for three or more SBM per week and CSBM per week in the naldemedine group versus the placebo group.

#### QOL Measures

The changes in EORTC QLQ-C15-PAL global QOL scale on day 7 and day 14 from day 1 were significantly higher in the naldemedine group than in the placebo group, indicative of better QOL (Table 2). The changes in PAC-QOL and PAC-SYM scores on day 14 from day 1 were significantly lower in the naldemedine group than in the placebo group, which indicated better constipation-related QOL with naldemedine.

Responses to EORTC QLQ-C15-PAL item 10 (constipation) with Very much, Quite a bit and A little were significantly lower with naldemedine than with placebo on days 7 and 14, albeit with insignificant differences on day 1.

#### OINV-Related Outcomes

The proportion of antiemetic drug use during the 72-hour period from days 1 to 3 was significantly lower with naldemedine than with placebo (Table 3). The proportion of patients with at least one episode of vomiting during the 72-hour period from days 1 to 3 was significantly lower in the naldemedine group than in the placebo group.

**TABLE 3.** Proportion of Patients Who Used Antiemetic Drugs Between Days 1 and 3, With At Least One Episode of Vomiting

End Point	Naldemedine Group (n = 48)	Placebo Group (n = 47)	Difference Between Groups (naldemedine-placebo)	*P*
Patients who used antiemetic drugs between days 1 and 3	10.6 (1.8-19.5)	51.1 (36.5-65.7)	–40.47 (23.41-57.53)	<.0001
Patients with at least one episode of nausea and/or vomiting				
Day 1	2.1 (0.0-6.3)	35.6 (21.6-49.5)	–33.4 (18.9-48.0)	<.0001
Day 2	6.4 (0.0-13.4)	46.6 (32.1-61.2)	–40.3 (24.1-56.5)	<.0001
Day 3	6.4 (0.0-13.4)	43.2 (28.6-57.8)	–36.8 (20.6-53.0)	<.0001

#### Safety Outcomes

There were no statistical differences between groups in the proportion of adverse events for abdominal distention, abdominal pain, diarrhea, bowel obstruction, and/or nausea. However, there was a significantly lower proportion of vomiting in patients treated with naldemedine. During the treatment period, no patient treated with naldemedine had diarrhea, nausea, or vomiting causally related to protocol treatment (Table 4).

**TABLE 4.** Adverse Events During the Protocol Treatment Period

Adverse Event	Naldemedine Group (n = 48), No. (%)					Placebo Group (n = 47), No. (%)					Group Comparison
	Grade 1-2	Grade 3	Grade 4	Grade 3 Over	Any Grade	Grade 1-2	Grade 3	Grade 4	Grade 3 Over	Any Grade	Mantel Test
All adverse events	—	—	—	—	14 (29.2)	—	—	—	—	29 (61.7)	—
Abdominal distension	0	0	0	0	0	2 (4.3)	0	0	0	2 (4.3)	0.1733
Abdominal pain	0	0	0	0	0	2 (4.3)	0	0	0	2 (4.3)	0.1508
Diarrhea	2 (4.2)	0	0	0	2 (4.2)	3 (6.4)	0	0	0	3 (6.4)	0.3450
Ileus	0	0	0	0	0	0	0	0	0	0	NC
Nausea	9 (18.8)	0	0	0	9 (18.8)	18 (38.3)	0	0	0	18 (38.3)	0.2119
Vomiting	6 (12.5)	0	0	0	6 (12.5)	18 (38.3)	1 (2.1)	0	1 (2.1)	19 (40.4)	0.0072
Hiccups	1 (2.1)	0	0	0	1 (2.1)	0	0	0	0	0	
Pruritus	0	0	0	0	0	1 (2.1)	0	0	0	1 (2.1)	
Weakness	1 (2.1)	0	0	0	1 (2.1)	0	0	0	0	0	
Somnolence	1 (2.1)	0	0	0	1 (2.1)	0	0	0	0	0	
Fatigue	1 (2.1)	0	0	0	1 (2.1)	1 (2.1)	0	0	0	1 (2.1)	
Anorexia	1 (2.1)	1 (2.1)	0	1 (2.1)	2 (4.2)	2 (4.3)	0	0	0	2 (4.3)	
Urinary retention	0	0	0	0	0	1 (2.1)	0	0	0	1 (2.1)	
Fever	1 (2.1)	0	0	0	1 (2.1)	0	0	0	0	0	
Edema	1 (2.1)	0	0	0	1 (2.1)	0	0	0	0	0	
Dysgeusia	0	0	0	0	0	1 (2.1)	0	0	0	1 (2.1)	
All adverse events related to protocol treatment	—	—	—	—	0	—	—	—	—	16 (34.0)	—
Diarrhea	2 (4.2)	0	0	0	2 (4.2)	2 (4.3)	0	0	0	2 (4.3)	NC
Nausea	9 (18.8)	0	0	0	9 (18.8)	11 (23.4)	0	0	0	11 (23.4)	NC
Vomiting	6 (12.5)	0	0	0	6 (12.5)	13 (27.7)	1 (2.1)	0	1 (2.1)	14 (29.8)	NC

Abbreviation: NC, not calculated.

Double-blind validation is summarized in Appendix 4.

## DISCUSSION

To our best knowledge, this is the first multicenter, double-blinded, randomized, placebo-controlled trial to demonstrate the preventive effect of naldemedine against constipation in patients with cancer receiving a strong opioid therapy.

A key finding is that prophylactic naldemedine resulted in a significantly higher proportion of patients with a BFI of <28.8, three or more SBM per week, and three or more CSBM per week on days 7 and 14 compared with placebo. Our finding is consistent with an observational study on opioid-naïve patients with cancer, which demonstrated BFI improved from baseline at 60 days in the oxycodone/naloxone group (–16 points), whereas BFI worsened in the prolonged-release oxycodone group (+13.8 points; between-group difference; *P* < .001).^21^ Our result was also consistent with both a systematic review of noncancer patients indicating that oxycodone/naloxone decreases the incidence of OIC^15^ and a previous post hoc analysis of a randomized open-label study which revealed that oxycodone/naloxone maintained normal BFI scores significantly better than oxycodone and morphine in opioid-naïve patients with noncancer pain.^16^ On the other hand, a randomized open-label study on patients with cancer failed to demonstrate the superiority of oxycodone/naloxone, but the intensity of constipation was measured as a secondary end point using unvalidated patient-reported bowel habit changes and laxative intake.^22^ Our study thus provides an evidence that prophylactic administration of naldemedine is effective in preventing constipation in patients with cancer receiving regularly dosed strong opioids therapy.

Next, our study demonstrated nominally significant better general QOL in the naldemedine group regarding differences between groups measured with the EORTC QLQ-C15-PAL global QOL scale, PAC-QOL, and PAC-SYM. A recent single-center, open-label, two-arm, phase II randomized, controlled trial report comparing naldemedine with magnesium oxide (MgO) for preventing constipation was consistent with our current results.^43^ That clinical trial revealed that naldemedine significantly prevented deterioration in PAQ-QOL and PAC-SYM scores from baseline to 2 weeks versus MgO.^43^ Thus, our results indicated that naldemedine may not only prevent constipation but also improve constipation-related QOL.

Finally, no naldemedine patient had diarrhea, nausea, or vomiting causally related to protocol treatment. This contrasts with a previous phase III, randomized, placebo-controlled trial that demonstrated that naldemedine for OIC management increased the risk of diarrhea, which led to study discontinuation.^12^ This difference may be from use of naldemedine after OIC, which causes diarrhea as a withdrawal symptom, whereas prophylactic use prevents withdrawal symptoms and reduces diarrhea. Thus, our study suggests that prophylactic administration of naldemedine may also preclude unnecessary diarrhea.

Of note, our study implies that naldemedine also has a preventive effect on OINV, consistent with a previous retrospective study that demonstrated significant lower OINV incidence with naldemedine within 2 days of opioid initiation versus control (36.0% *v* 47.2%; *P* = .046).^18^ If naldemedine is prophylactic against OINV, a single dose of naldemedine could prevent both constipation and nausea without dose adjustment, improving patient compliance. Further randomized, controlled trials focusing primarily on the prophylactic effect of naldemedine on OINV will be promising.

Clinically, for OIC prevention, naldemedine and standard laxatives have benefits and disadvantages. Naldemedine benefits are evidence from clinical trials and freedom from cumbersome daily adjustments, but availability or expense may vary by country. Routine laxatives, meanwhile, are widely available and inexpensive, but efficacy evidence is limited and daily, monitored self-adjustment is required.^6^ Although comparative studies are needed to clarify clinical benefits for each treatment, individualized medication to meet personal goals is recommended.

Limitations must be acknowledged. First, before enrollment, approximately 30% of patients had been using rescue laxatives and 20% of patients had been using strong opioids, albeit for rescue use only. Despite this, the proportions were well balanced, and we believe this was unlikely to affect conclusions. Second, although the majority of enrolled patients were outpatients with abdominal cancer, our study enrolled a heterogeneous population of patients with cancer with different types, stages, and treatments of cancer, which might affect the result as confounding factors. Third, our study had a relatively short treatment period, which may not capture long-term effects and safety of naldemedine for OIC prevention in patients with cancer receiving regularly dosed opioids therapy. Thus, examining the long-term efficacy of OIC when naldemedine is administered prophylactically is necessary. Fourth, the study was conducted only in a Japanese population in specific settings (ie, palliative care consultation in acute hospital settings). Thus, the generalizability of the results might be limited with regard to other ethnic populations and settings. Fifth, BFI >28.8 score is not exactly the same as a diagnosis of OIC on the basis of Rome IV criteria but rather indicates clinically significant constipation. We do not believe setting the threshold value of 28.8 affects our conclusion because all end points, including analyses of mean BFI values, achieved the same results. Sixth, this study did not compare the efficacy of naldemedine and traditional laxatives, as well as other PAMORAs, and this should be investigated in a further study. Seventh, cost-effectiveness was not explored in this study and should be investigated in future studies. Finally, the lactose used in the intervention and placebo drugs in this study might have affected bowel movements, especially in lactose-intolerant individuals.

In conclusion, naldemedine prevented constipation and improved constipation-related QOL, with possible preventive effect on OINV, in patients with cancer starting regularly dosed opioids therapy.

## Data Availability

The authors declare that all data supporting the findings of this study are available within the Article and its appendix. Researchers can apply for data by submitting a proposal to the corresponding author.

## APPENDIX 1. RATIONALE FOR CHOOSING A PLACEBO-CONTROLLED DESIGN

To our understanding, there are no double-blind, randomized, placebo-controlled trials to determine the efficacy of conventional laxatives in patients initiating regularly dosed opioids therapy. Empirical evidence is limited to comparisons of laxatives, cohort studies, or analyses of secondary end points in randomized controlled trials to confirm the analgesic efficacy of oxycodone/naloxone.^9,21,22^ Thus, even if a comparison of naldemedine and conventional laxatives were to reveal that naldemedine is superior to the laxative, we could not reasonably conclude that naldemedine is more effective than placebo in preventing opioid-induced constipation (ie, both could possibly be inferior to placebo). We therefore chose a placebo-controlled design.

## APPENDIX 2. RATIONALE FOR USE OF RESCUE LAXATIVES

This rescue laxatives regimen was determined on the basis of clinical trials on chronic constipation, where there is a wealth of clinical research.^26^ Since several practice guidelines recommend titrating laxatives, such as senna, to meet individual treatment goals,^2,3^ these rescue laxatives were considered ethically justified to ensure opioid-induced constipation prophylactic efficacy in the placebo group. Informed consent explanations included this background.

## APPENDIX 3. SAMPLE SIZE CALCULATION

Since no intervention studies on opioid-induced constipation (OIC) prevention using Bowel Function Index measure existed during study design, we calculated sample sizes on the basis of similar observational reports.^41^ A multicenter, prospective observational study reported that 65% of patients with cancer who started regular opioid prescriptions without prophylactic laxatives developed OIC on the basis of Rome IV criteria and 32% of patients with prophylactic laxatives developed OIC.^41^

Assuming a constipation incidence of 65% in the control group and 35% in the naldemedine group in this trial, with a significance level of 5% on two-sided tests and a power of 80%, the required patient number becomes 43 in each group, for a total of 86 patients. Assuming a dropout rate of 10%, the enrolled patient target number was set at 100.

## APPENDIX 4. DOUBLE-BLIND VALIDATION

Of the naldemedine group patients (n = 48), 18.8% (n = 9) thought the drug was naldemedine, 22.9% (n = 11) thought it was placebo, and 58.3% (n = 28) were unsure. Within the placebo group (n = 43), 14.0% (n = 6) thought the drug was placebo, 11.6% (n = 5) thought it was naldemedine, and 72.1% (n = 31) were unsure. The percentage of patients' perceived drug groups that matched their actual drug groups was 16.5% (n = 15) and the percentage of physician matching was 19.8% (n = 18).

**TABLE A1.** Detailed Inclusion and Exclusion Criteria

Definition
Inclusion criteria
Patients with cancer starting regular strong opioid (morphine, oxycodone, hydromorphone) medication for the first time for cancer pain
Age 20 years or older (at the time of obtaining consent)
Patients who can take oral medications, meals, and beverages
Patients who are considered capable of self-documentation in the patient diary (proxy documentation in the patient diary is acceptable if the patient is capable of self-assessment)
Patients who are not expected to experience a rapid change in their cancer condition during the protocol treatment period
Patients who received sufficient explanation, have an understanding of the study, and who freely gave their written consent to participate
Exclusion criteria
Patients with GI obstruction (actual or suspected) or patients with a history of GI obstruction and a high risk of recurrence
Patients who have undergone surgery, radiotherapy, or procedures affecting GI function (eg, nerve blocks) within 14 previous days before the date of enrollment or who will undergo such procedures within the protocol treatment period
Patients with medically significant clinical laboratory values, electrocardiograms, or physical examinations who are judged as inappropriate to participate in the study
Patients who previously took or are currently taking naldemedine
Patients who had severe diarrhea (more than seven times a day) within 7 previous days before the date of enrollment or who underwent stool extraction for constipation
Patients who have used opioid patches or opioid injections within 7 previous days before the date of enrollment
Patients who are undergoing cancer drug therapy that is expected to affect defecation within 14 days (retrospectively) from the initial enrollment date or who are scheduled to undergo such therapy within the protocol treatment period

**TABLE A2.** Detailed End Points

Definition
The primary end point
The proportion of patients with a BFI of <28.8 on day 14
Secondary end points
The proportion of patients with a BFI of <28.8 on day 7
The amount and rate of change from day 1 in BFI for days 7 and 14
The proportion of patients with three or more SBM (the number of defecations, excluding those within 24 hours after rescue laxative administration) per week on days 7 and 14 (frequency)
The proportion and number of patients with three or more complete SBM (the number of bowel movements without a residual stool feeling, excluding bowel movements within 24 hours after rescue laxatives were administered) on days 7 and 14
Bowel movement during defecation (yes/no), squeezing during each defecation (not at all/just a little/moderately/quite a lot/very much), residual feeling during each defecation (yes/no)
Overall bowel movements (four levels: dissatisfied, somewhat dissatisfied, somewhat satisfied, and satisfied) in the last week on days 1, 7, and 14
EORTC QLQ-C15-PAL and subscale scores for days 1, 7, and 14
The proportion of patients who responded to EORTC QLQ-C15-PAL item 9 (nausea) on days 1, 7, and 14
The proportion of patients who responded to EORTC QLQ-C15-PAL item 10 (constipation) on days 1, 7, and 14
Changes in bowel movements between days 1 and 7 and between days 1 and 14
The Japanese version of the PAC-QOL and the PAC-SYM on days 1 and 14
Amount of change in PAC-QOL on days 1 and 14, plus amount of change in PAC-SYM on days 1 and 14
The proportion of patients who had at least one episode of vomiting during the 72-hour period from days 1-3
The proportion of patients who used antiemetic drugs during the 72-hour period from days 1-3
The number of diarrhea episodes that occurred during administration of the study drug
The number of times rescue laxatives were used
Adverse events that occurred during study drug administration
Double-blind validation

Abbreviations: BFI, Bowel Function Index; EORTC QLQ-C15-PAL, European Organization for Research and Treatment of Cancer Quality of Life Questionnaire Core 15 Palliative; PAC-QOL, Patient Assessment of Constipation Quality of Life questionnaire; PAC-SYM, Patient Assessment of Constipation Symptoms questionnaire; SBM, spontaneous bowel movements.

**TABLE A3.** Use Frequency of Rescue Laxatives

Day	Naldemedine Group, No. (%) (n = 48)	Placebo Group, No. (%) (n = 47)	*P*
Day 1			
0	47 (97.9)	44 (93.6)	
1	1 (2.1)	1 (2.1)	
2	0	0	
3	0	0	
4	0	0	
Missing	0	2 (4.3)	
Day 2			
0	46 (95.8)	39 (83.0)	
1	1 (2.1)	6 (12.8)	
2	0	0	
3	0	0	
4	0	0	
Missing	1 (2.1)	2 (4.3)	
Day 3			
0	45 (93.8)	37 (78.7)	
1	2 (4.2)	7 (14.9)	
2	0	0	
3	0	0	
4	0	0	
Missing	1 (2.1)	3 (6.4)	
Day 4			
0	45 (93.8)	33 (70.2)	
1	2 (4.2)	11 (23.4)	
2	0	0	
3	0	0	
4	0	0	
Missing	1 (2.1)	3 (6.4)	
Day 5			
0	46 (95.8)	37 (78.7)	
1	1 (2.1)	6 (12.8)	
2	0	0	
3	0	0	
4	0	0	
Missing	1 (2.1)	4 (8.5)	
Day 6			
0	46 (95.8)	36 (76.6)	
1	1 (2.1)	7 (14.9)	
2	0	0	
3	0	0	
4	0	0	
Missing	1 (2.1)	4 (8.5)	
Day 7			
0	43 (89.6)	37 (78.7)	
1	4 (8.3)	6 (12.8)	
2	0	0	
3	0	0	
4	0	0	
Missing	1 (2.1)	4 (8.5)	
Day 8			
0	46 (95.8)	36 (76.6)	
1	1 (2.1)	6 (12.8)	
2	0	0	
3	0	0	
4	0	0	
Missing	1 (2.1)	5 (10.6)	
Day 9			
0	43 (89.6)	33 (70.2)	
1	4 (8.3)	9 (19.1)	
2	0	0	
3	0	0	
4	0	0	
Missing	1 (2.1)	5 (10.6)	
Day 10			
0	45 (93.8)	35 (74.5)	
1	1 (2.1)	6 (12.8)	
2	1 (2.1)	1 (2.1)	
3	0	0	
4	0	0	
Missing	1 (2.1)	5 (10.6)	
Day 11			
0	47 (97.9)	44 (93.6)	
1	1 (2.1)	1 (2.1)	
2	0	0	
3	0	0	
4	0	0	
Missing	0	2 (4.3)	
Day 12			
0	44 (91.7)	36 (76.6)	
1	3 (6.3)	6 (12.8)	
2	0	0	
3	0	0	
4	0	0	
Missing	1 (2.1)	5 (10.6)	
Day 13			
0	46 (95.8)	36 (76.6)	
1	1 (2.1)	6 (12.8)	
2	0	0	
3	0	0	
4	0	0	
Missing	1 (2.1)	5 (10.6)	
Day 14			
0	46 (95.8)	38 (80.9)	
1	2 (4.2)	4 (8.5)	
2	0	0	
3	0	0	
4	0	0	
Missing	0	5 (10.6)	
Total, n	48	45^a^	
Mean (SD)	0.6 (1.5)	2.0 (3.0)	.0059

Abbreviation: SD, standard deviation.

^a^ The data about use of rescue laxatives were lacking in two patients.
